# Myelitis of the Anterior Horns

**Published:** 1877-07

**Authors:** 


### Books Reviewed.

*Myelitis of the Anterior Horns, or Spinal Paralysis of the Adult and Child.* By E. C. Seguin, M. D., Clinical Professor of Diseases of the Mind and Nervous System, in the College of Physicians and Surgeons, New York: G. P. Putnam's Sons, 182 Fifth Avenue, 1877.

This volume is elegant in appearance, clear and forcible in expression, and an important addition to the literature of the subject of which it treats. Forty-five clinical cases have been gathered from the practice of some of the leading physicians of this and other countries. After giving their history in detail, the author presents the symptoms analytically and synthetically studied.

A neatly arranged table of autopsies of twenty-nine cases of infantile spinal paralysis is an interesting feature of the book. Diagnosis, Aetiology Treatment and Prognosis, each forms the subject of a brief chapter. Exposure to cold and dampness, and acute contagious diseases are given as chief among the known causes of "Myelitis of the anterior horns." The pathological anatomy of the disease has been rigidly investigated, and prognosis and treatment as given by the author, are rational sequences of this antecedent.

